# Spontaneous cholecystocutaneous fistula: a rare complication of gallbladder disease

**DOI:** 10.1590/S1516-31802006000400012

**Published:** 2006-05-04

**Authors:** Ruy Jorge Cruz, Jorge Nahas, Luiz Francisco Poli de Figueiredo

**Keywords:** Digestive system fistula, Abscess, Tomography, Cholecystectomy, Surgery, Fístula do sistema digestório, Abscesso, Tomografia, Colecistectomia, Cirurgia

## Abstract

**CONTEXT:**

Spontaneous cholecystocutaneous abscess or fistula is an extremely uncommon complication secondary to cholecystitis. Over the past 50 years fewer than 20 cases of spontaneous cholecystocutaneous fistulas have been described in the medical literature. We here report a case of subcutaneous gallstone as a rare clinical presentation of the already uncommon cholecystocutaneous fistula.

**CASE REPORT:**

An 81-year-old man presented with a large subcutaneous abscess in the right subcostal area with surrounding cellulitis and crepitus. An abdominal computed tomography scan showed two subcutaneous gallstones and communication between the abscess and the gallbladder. Cholecystectomy was performed and the abdominal wall abscess was drained externally. This case report demonstrates that maintaining a high degree of suspicion of this rare entity is helpful in achieving correct preoperative diagnosis, and that computed tomography scan should be performed in all cases of unexplained abdominal wall suppuration or cellulitis.

## Resumo

**CONTEXTO:**

A fístula e/ou o abscesso colecistocutâneo são complicações extremamente raras da colecistopatia crônica calculosa. Nos últimos 50 anos, menos de 20 casos de fistulas colecistocutâneas foram descritos na literatura médica. Nós descrevemos um caso de cálculos biliares no tecido subcutâneo como rara apresentação clínica de fístula biliar.

**RELATO DO CASO:**

Paciente de 81 anos deu entrada no serviço de emergência apresentando um grande abscesso na região de hipocôndrio direito. A tomografia computadorizada de abdome demonstrou a presença de dois cálculos no tecido celular subcutâneo e a comunicação da vesícula biliar com a parede abdominal. O paciente foi submetido a uma colecistectomia e drenagem externa do abscesso da parede abdominal. Este caso demonstra que um alto grau de suspeita desta rara complicação da colecistopatia crônica calculosa é fundamental para o correto diagnóstico pré-operatório, e a tomografia computadorizada deve ser realizada rotineiramente em todos os casos de abscessos de parede abdominal de origem indeterminada.

## INTRODUCTION

Spontaneous cholecystocutaneous abscess or fistula is an extremely uncommon complication of gallbladder disease that has been known since the time of Thilesius in 1670. Since 1900, 72 cases have been reported in medical literature. However, over the past 50 years fewer than 20 cases of spontaneous cholecystocutaneous fistulas have been described. The marked decrease in the incidence of this complication is probably associated with the advent of contemporary diagnostic methods, broad-spectrum antibiotic therapy, and early and effective surgical management of biliary tract disease.^1,2^ We here report a case of subcutaneous gallstones as a rare clinical presentation of the already uncommon cholecystocutaneous fistula.

## CASE REPORT

An 81-year-old man was admitted to our Emergency Department complaining of a right upper abdominal pain that had lasted for 28 days, concomitant to the development of a right subcostal mass. The patient presented ischemic myocardiopathy with left bundle block on electrocardiogram. There was no previous history of abdominal pain, local trauma, dyspepsia or diabetes. On admission, the patient was in a good clinical condition, with a heart rate of 100 beats/min and systolic blood pressure of 110 mmHg, and he was afebrile and anicteric.

Abdominal examination revealed a 10-cm abdominal wall abscess in the right subcostal area with surrounding cellulitis, crepitus and tenderness, without guarding. The white blood cell count was 8,800/mm^3^, with 7% monocytes, 77% segments and 1% bands. The other laboratory data were unremarkable, including normal hemoglobin, bilirubin, alkaline phosphatase, amylase and glucose levels. Radiographic studies revealed the presence of gas and two ring-shaped images with signs of calcification in the subcutaneous space.

**Figure 1:**
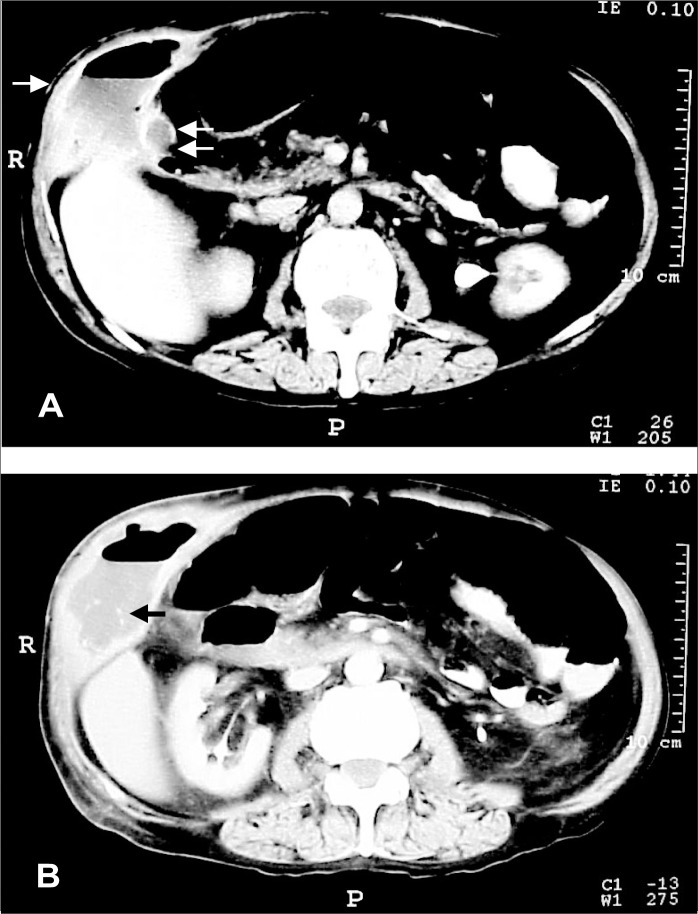
Abdominal computed tomography scan: (A) subcutaneous abscess in the abdominal wall with inner gas (single arrow), and the gallbladder (double arrow); (B) gallstones in the subcutaneous space (arrow).

Abdominal ultrasound was not performed because of local pain at the abscess site. An abdominal computed tomography scan clearly showed two subcutaneous gallstones and communication between the abscess and the gallbladder (Figure 1 A and B).

The patient underwent median laparotomy; the contracted gallbladder was freed from inflammatory adhesions to the abdominal wall abscess site; and cholecystectomy with intra-operative cholangiography was performed. Three multifaceted gallstones were extracted from the subcutaneous layer, and the subcutaneous abscess was drained externally, before abdominal closure.

Histopathological analysis revealed chronic cholecystitis with no evidence of malignancy. The immediate postoperative course was uneventful; however, three days later, the patient presented an acute onset of chest pain, dyspnea and refractory shock from massive pulmonary embolism, in spite of prophylaxis for deep vein thrombosis. He died in the intensive care unit 12 hours later.

## DISCUSSION

External biliary fistulas or abscesses rarely occur spontaneously as a result of intrahepatic abscess (pyogenic or parasitic), necrosis or perfo-ration of the gallbladder, or other inflamma tory process involving the biliary tree. Most cholecystocutaneous abscesses or fistulas are postoperative complications of liver and biliary tract surge ry or trauma. We have reported a rare case of spon taneous gallbladder perforation resul ting in cholecystocutaneous abscess with multi ple gallstones inside it. To our knowledge, there has not been any other case of cholecystocutaneous abscess or fistula presenting with multiple subcuta neous gallstones in the English-language medical literature since 1949.^1^

In a recent review, Kaminsky reported on the frequency of biliary fistula to the gastrointestinal tract. The great majority of the fistulas occur with connection to the duodenum (60%), followed by the colon (24%), stomach (6%) and choledochal duct (5%). In this series, cholecystocutaneous abscesses or fistulas accounted for only 2% of all the cases. Generally, the fistulas or abscesses appear in the right upper quadrant, although other locations such as the epigastrium, umbilical area, right groin and even the gluteal region have also been described.^1–3^

Cholecystocutaneous fistulas are often the result of neglected biliary tract disease. Patients with this complication usually do not report a distinct episode of acute cholecystitis in their history, since this would have brought such a patient to seek medical attention sooner. The patients are usually women over the age of 60. However, cases have been documented in patients as young as 24 years old.^2^

The pathophysiology of this condition has been associated with increased pressure in the gallbladder, secondary to cystic duct obstruction, either caused by a calculus or neoplasia. The increase in intraluminal pressure leads to impairment of the blood flow and lymph supply to the gallbladder, thus causing mural necrosis and perforation. Perforation can occur as 1) acute-free perforation leading to peritonitis, 2) subacute perforation resulting in an abscess around the gallbladder, or 3) chronic perforation with the formation of an internal or external biliary fistula. These fistulas, as presented in this case, frequently arise from the fundus of the gallbladder.^1,2^ The state preceding spontaneous rupture has been termed “empyema necessitatis” by Nayman.^4^ This term essentially describes a “burrowing abscess” of the abdominal wall as a result of gallbladder inflammation.

Two different approaches are considered for this unusual complication of gallbladder disease. In the past, external drainage of the abscess and antibiotics were used for enabling biliary drainage and sepsis control. However, this approach has been associated with biliary fistula formation when the cystic duct is patent, which would cause the discomfort of an openly draining wound, besides requiring an additional, definitive surgical procedure. Therefore, two-stage surgical treatment has been recommended only for septic patients in a poor clinical condition.^1,2^ A single-stage procedure like the one we performed is considered to be the treatment of choice. Recently, Kumar described the first case of successful single-stage laparoscopic management of a cholecystocutaneous fistula, which suggests that this approach is feasible.^5^

This case report demonstrates that maintaining a high degree of suspicion of this rare entity is helpful in achieving correct preoperative diagnosis, and that computed tomography scan should be performed in all cases of unexplained abdominal wall suppuration or cellulitis. However, continued advances in noninvasive diagnosis, and widespread of elective and emergency cholecystectomy will eventually put an end to this exceedingly rare complication of common gallbladder disease.
